# Immune-Related Adverse Events and Survival Among Patients With Metastatic NSCLC Treated With Immune Checkpoint Inhibitors

**DOI:** 10.1001/jamanetworkopen.2023.52302

**Published:** 2024-01-18

**Authors:** Sarah Cook, Vanessa Samuel, Daniel E. Meyers, Igor Stukalin, Ishjot Litt, Randeep Sangha, Don G. Morris, Daniel Y. C. Heng, Aliyah Pabani, Michelle Dean, Vishal Navani

**Affiliations:** 1 Department of Medical Oncology, University of Calgary, Calgary, Alberta, Canada; 2 Department of Medical Oncology, University of Manitoba, Winnipeg, Manitoba, Canada; 3 Cross Cancer Institute, Edmonton, Alberta, Canada; 4 Tom Baker Cancer Centre, Calgary, Alberta, Canada; 5 Department of Oncology, Johns Hopkins University, Baltimore, Maryland

## Abstract

**Importance:**

Immune-related adverse events (irAEs) secondary to immune checkpoint inhibitor (ICI) therapy reportedly improve overall survival (OS) in patients with non–small cell lung cancer (NSCLC). However, studies have been small and the association between irAE severity and OS remains poorly defined.

**Objective:**

To examine the association between irAEs and their severity with OS in patients with locally advanced or metastatic NSCLC receiving ICIs.

**Design, Setting, and Participants:**

This retrospective observational cohort study included patients with NSCLC receiving ICIs between March 1, 2014, and November 30, 2021, with follow-up until March 31, 2023. Data analysis was completed April 26, 2023. The Alberta Immunotherapy Database, a provincial, multicenter cohort, was used to capture data from patients receiving ICIs in Alberta, Canada. Participants included 803 patients 18 years or older who received at least 1 cycle of ICI (alone or with chemotherapy), agnostic to treatment line.

**Exposure:**

Developing an irAE mandating delay or discontinuation of ICI therapy and/or systematic corticosteroids for management of toxic effects (hereinafter referred to as clinically meaningful irAEs).

**Main Outcomes and Measures:**

The primary outcome was association between irAEs and OS according to Kaplan-Meier analysis. Clinically meaningful irAEs were identified. Patients with poor prognosis (survival <3 months) who may have died prior to irAE development were excluded from OS analysis, mitigating immortal time bias. Adjusted Cox proportional hazards regression analyses ascertained variables associated with OS.

**Results:**

Among the 803 patients included in the analysis, the median age of patients with irAEs was 69.7 (IQR, 63.1-75.2) years and the median age of those without irAEs was 67.5 (IQR, 60.4-73.3) years, with comparable sex distribution (139 of 295 men [47.1%] and 156 of 295 women [52.9%] with irAEs vs 254 of 505 men [50.3%] and 251 of 505 women [49.7%] without irAEs). Mitigating immortal time bias (n = 611), irAEs were associated with OS (median OS with irAEs, 23.7 [95% CI, 19.3-29.1] months; median OS without irAEs, 9.8 [95% CI, 8.7-11.4] months; *P* < .001). No OS difference was associated with treatment in hospital vs as outpatients for an irAE (median OS, 20.8 [95% CI, 11.7-30.6] vs 25.6 [95% CI, 20.1-29.8] months; *P* = .33). Developing irAEs remained associated with OS in the total cohort after Cox proportional hazards regression with known prognostic characteristics (hazard ratio, 0.53 [95% CI, 0.40-0.70]; *P* < .001).

**Conclusions and Relevance:**

In this cohort study of 803 patients with locally advanced or metastatic NSCLC receiving ICIs, developing a clinically meaningful irAE was associated with improved OS. This association was not compromised by hospitalization for severe toxic effects. Whether and how ICI therapy resumption after an irAE is associated with OS warrants further study.

## Key Points

**Question:**

Are immune-related adverse events associated with improved overall survival in patients with locally advanced or metastatic non–small cell lung cancer receiving immune checkpoint inhibitor (ICI) therapy?

**Findings:**

In this cohort study of 803 patients, developing an immune-related adverse event mandating delay or discontinuation of ICI therapy and/or systemic corticosteroids for management of toxic effects was associated with significantly improved median overall survival. This association was not compromised by hospitalization for management of toxic effects of severe immune-related adverse events.

**Meaning:**

These findings suggest that immune-related adverse events are associated with improved overall survival in patients receiving ICI therapy, even in the context of hospitalization for management of toxic effects.

## Teaser

This cohort study investigates the association of immune-related adverse events with overall survival among patients receiving immune checkpoint inhibitors (ICIs) to treat metastatic non–small cell lung cancer (NSCLC).

## Introduction

Globally, lung cancer remains the leading cause of cancer-related death.^1^ Improvements are being made, however, and overall mortality has declined over the past decade. This decline has largely been driven by treatment advances in non–small cell lung cancer (NSCLC), with immune checkpoint inhibitors (ICIs) improving clinical outcomes.^2^ Several clinical trials examining ICIs as monotherapy^3,4,5,6,7^ or in combination with chemotherapy,^8,9,10,11^ angiogenesis inhibitors,^12^ and a second ICI agent^13,14,15^ have demonstrated improved time to event end points and response rates. The treatment landscape in metastatic NSCLC has thus been transformed.

It is well established that although ICIs improve clinical outcomes in NSCLC, they are complicated by immune-related adverse events (irAEs), the incidence of which varies by study setting, patient selection, treatment parameters, and irAE grading.^16,17,18,19,20,21,22,23,24,25,26,27,28,29,30,31,32,33,34,35,36,37,38,39,40,41,42,43,44,45,46,47,48^ There is growing recognition across solid malignant neoplasms that irAEs affect overall survival (OS). In advanced or metastatic NSCLC, the data suggest that developing an irAE is associated with improved OS. However, the ability to form firm conclusions has been limited by data predominantly arising from small studies, focusing largely on nivolumab monotherapy, and with the clinical effect of irAEs on management decisions poorly described.^31,32,33,49,50,51^ It has also been challenging to form firm conclusions on the association between irAE severity and OS in advanced or metastatic NSCLC, as published results are conflicting.^31,32,33,52^

We examined a contemporary multicenter cohort of patients with locally advanced or metastatic NSCLC treated with ICI alone or combined with chemotherapy, agnostic to treatment line. We investigated the association of survival outcomes with (1) irAEs mandating delay or discontinuation of ICI therapy and/or systemic corticosteroids for management of toxic effects (hereinafter termed *clinically meaningful irAEs*) and (2) hospitalization for irAEs.

## Methods

### Study Design

In this cohort study, we examined data from the Alberta Immunotherapy Database, a contemporary provincial multicenter observational cohort nested in routine clinical practice. Using a standardized template, the Alberta Immunotherapy Database captures information from patients treated with ICI, including baseline demographic characteristics (age and gender), tumor histology, and clinical, laboratory, and imaging data.^53^ Study approval was obtained from the Health Research Ethics Board of the Alberta Cancer Committee, which waived the need for patient consent given the low-risk, deidentified, retrospective nature of the study. The study followed the Strengthening the Reporting of Observational Studies in Epidemiology (STROBE) reporting guideline.

Patients were included if they had metastatic NSCLC or unresectable locally advanced disease ineligible for curative intent chemoradiotherapy and/or durvalumab. Additional study inclusion criteria were age 18 years or older and completion of at least 1 cycle of ICI therapy (atezolizumab, nivolumab, or pembrolizumab alone or combined with chemotherapy) of noncurative intent, agnostic to treatment line. Age and gender are included in the baseline demographic data captured through the Alberta Immunotherapy Database, which relies on information collected through our electronic health records system and currently does not include race or ethnicity.

Patients received treatment between March 1, 2014, and November 30, 2021. The date of last follow-up was March 31, 2023. Study investigators assessed the objective imaging response using Response Evaluation Criteria in Solid Tumours, version 1.1, prior to data analysis.^54^ Where treatment duration data were missing, patients were excluded from survival analysis.

### Outcomes of Interest

**Figure 1.**
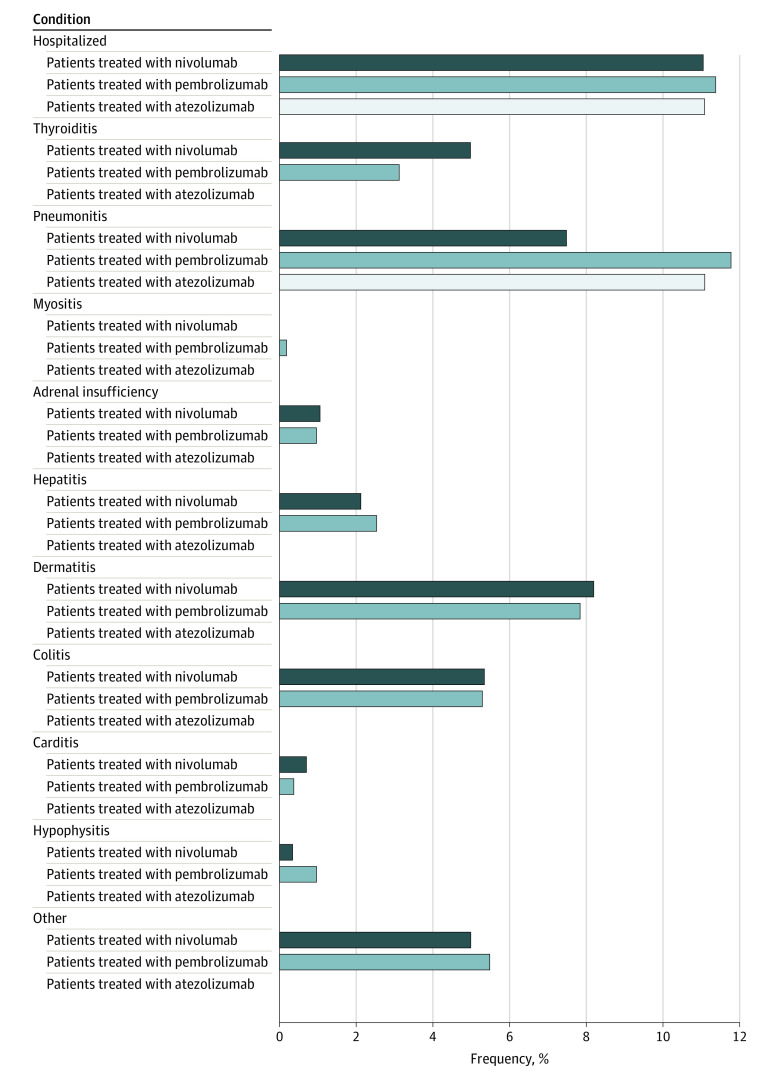
Relative Frequency and Type of Clinically Meaningful Immune-Related Adverse Event by Agent Received

We investigated patients with locally advanced or metastatic NSCLC who met the study inclusion criteria. Clinically meaningful irAEs were identified. As detailed in Figure 1, irAEs were categorized as thyroiditis, pneumonitis, myositis, adrenal insufficiency, hepatitis, dermatitis, colitis, carditis, and hypophysitis. Less commonly occurring irAEs were collectively categorized as “other.” Clinically meaningful irAEs were defined as mandating delay or discontinuation of ICI therapy and/or immunosuppression (oral or intravenous corticosteroids) for management of toxic effects. Patients who did and did not develop irAEs during ICI therapy (alone or combined with chemotherapy) were compared. Our primary end point was the association between developing a clinically meaningful irAE and overall survival (OS). We further examined variables associated with OS, including baseline characteristics (eg, demographic, tumor histology, mutation status, laboratory parameters), irAE development, and hospitalization for irAE management. Our secondary end point was the association between developing a clinically meaningful irAE and time to next treatment (TTNT).

Electronic medical records were reviewed to identify irAEs during ICI therapy. Only clinically meaningful irAEs as described above were recorded. Where multiple irAEs occurred in the same patient, the most clinically meaningful irAE was included.

### Statistical Analysis

Statistical analysis was completed April 26, 2023. Both the χ^2^ test and the Fisher exact test were used to identify baseline characteristics that were prognostic factors associated with developing an irAE. To mitigate immortal time bias from patients with a poor prognosis who may have died before developing an irAE, a 12-week cutoff was used for OS and TTNT analyses. This cutoff was selected because it corresponds to the median time (3.4 [IQR, 1.4-8.4] months) to irAE development in our study. Thus, patients dying within 12 weeks of ICI therapy initiation were excluded from Kaplan-Meier analysis (compared using a log-rank test). We defined OS as time from ICI therapy initiation to date of death or last censored follow-up. We defined TTNT as time from ICI initiation to starting the next line of systemic therapy, death, or last censored follow-up. A multivariable Cox proportional hazards regression model was applied to OS and TTNT analysis to adjust for significant differences in baseline characteristics, including treatment line and use of ICI alone or combined with chemotherapy.

Where data were missing, the case deletion method was used. All statistical tests were 2 sided. Results were considered statistically significant at *P* ≤ .05. SAS Cloud of Academics, version 9.4 (SAS Institute Inc) was used for analysis.

## Results

### Characteristics of Population, Disease, and Treatments Received

A total of 803 patients with locally advanced or metastatic NSCLC treated with ICIs with noncurative intent during the period of interest were included in the analysis. A clinically meaningful irAE was confirmed in 297 patients. The median follow-up was 51.9 (95% CI, 49.4-56.2) months. Patient baseline characteristics are summarized in Table 1. The median age of patients who developed an irAE was 69.7 (IQR, 63.1-75.2) years compared with 67.5 (IQR, 60.4-73.3) years among those who did not. Sex distribution was comparable (139 of 295 men [47.1%] and 156 of 295 women [52.9%] with irAEs vs 254 of 505 men [50.3%] and 251 of 505 women [49.7%] without irAEs). Patients who developed an irAE had a median 10 (IQR, 4-23) ICI cycles compared with 4 (IQR, 2-9) among those who did not. The following baseline characteristics were associated with developing an irAE: 60 years or older (245 of 296 [82.8%] vs 388 of 505 [76.8%]; *P* = .05), Eastern Cooperative Oncology Group (ECOG) performance status 0 (56 of 294 [19.0%] vs 46 of 500 [9.2%]; *P* < .001), high expression of programmed cell death ligand 1 (PD-L1) (160 of 240 [66.7%] vs 230 of 404 [56.9%]; *P* = .05), no metastases to bone (215 of 296 [72.6%] vs 322 of 503 [64.0%]; *P* = .01), derived neutrophil lymphocyte ratio (DNLR) 3 or less (181 of 260 [69.6%] vs 242 of 452 [53.5%]; *P* < .001), and levels within reference range for hemoglobin (175 of 265 [66.0%] vs 264 of 457 [57.8%]; *P* = .03), albumin (189 of 240 [78.8%] vs 263 of 412 [63.8%]; *P* < .001), and lactate dehydrogenase (152 of 212 [71.7%] vs 217 of 358 [60.6%]; *P* = .007). Developing an irAE was not associated with patient sex, tumor histology, or the presence of an underlying driver mutation.

**Table 1.** Baseline Characteristics of Patients With NSCLC With or Without irAEs

Characteristics	Patient group (n = 803)^a^		*P* value^b^
	Without irAEs (n = 506)	With irAEs (n = 297)	
Age, y			
>60	388/505 (76.8)	245/296 (82.8)	.05
≤60	117/505 (23.2)	51/296 (17.2)	
Median (IQR)	67.5 (60.4-73.3)	69.7 (63.1-75.2)	NA
BMI, median (IQR)	25.0 (22.0-28.2)	25.9 (23.0-30.3)	NA
Sex			
Men	254/505 (50.3)	139/295 (47.1)	.39
Women	251/505 (49.7)	156/295 (52.9)	
ECOG performance status at ICI therapy start^c^			
0	46/500 (9.2)	56/294 (19.0)	<.001
1	273/500 (54.6)	187/294 (63.6)	
2	156/500 (31.2)	43/294 (14.6)	
3	25/500 (5.0)	8/294 (2.7)	
Hemoglobin level			
Within reference range	264/457 (57.8)	175/265 (66.0)	.03
Below lower limit of reference	193/457 (42.2)	90/265 (34.0)	
Lactate dehydrogenase			
Within reference range	217/358 (60.6)	152/212 (71.7)	.01
Above upper limit of reference	141/358 (39.4)	60/212 (28.3)	
Albumin			
Within reference range	263/412 (63.8)	189/240 (78.8)	<.001
Below lower limit of reference	149/412 (36.2)	51/240 (21.3)	
DNLR			
≤3	242/452 (53.5)	181/260 (69.6)	<.001
>3	210/452 (46.5)	79/260 (30.4)	
Histology			
Adenocarcinoma	362/504 (71.8)	213/296 (72.0)	.92
Squamous cell carcinoma	106/504 (21.0)	64/296 (21.6)	
Other	36/504 (7.1)	19/296 (6.4)	
PD-L1 expression, %			
<1	86/404 (21.3)	42/240 (17.5)	.05
1-49	88/404 (21.8)	38/240 (15.8)	
>50	230/404 (56.9)	160/240 (66.7)	
*EGFR* mutation			
Present	20/403 (5.0)	7/227 (3.1)	.26
Absent	383/403 (95.0)	220/227 (96.9)	
*ALK* mutation			
Present	1/387 (0.3)	0/225	.45
Absent	386/387 (99.7)	225/225 (100)	
*BRAF* mutation			
Present	0/93	2/70 (2.9)	.10
Absent	93/93 (100)	68/70 (97.1)	
*KRAS* mutation			
Present	45/97 (46.4)	35/70 (50.0)	.65
Absent	52/97 (53.6)	35/70 (50.0)	
*ROS-1* mutation			
Present	7/43 (16.3)	2/18 (11.1)	.60
Absent	36/43 (83.7)	16/18 (88.9)	
*PIK3CA* mutation			
Present	1/57 (1.8)	1/43 (2.3)	.84
Absent	56/57 (98.2)	42/43 (97.7)	
*MET* mutation			
Present	1/1 (100)	2/4 (50.0)	.36
Absent	0/1	2/4 (50.0)	
Metastatic site^d^			
Lung	320/504 (63.5)	130/296 (43.9)	.04
Bone	181/503 (36.0)	81/296 (27.4)	.01
Brain	86/502 (17.1)	39/296 (13.2)	.14
Liver	106/503 (21.1)	50/296 (16.9)	.15
Adrenal	82/505 (16.2)	47/296 (15.9)	.89
ICI agent			
Nivolumab	181/502 (36.1)	99/296 (33.4)	.18
Pembrolizumab	313/502 (62.4)	196/296 (66.2)	
Atezolizumab	8/502 (1.6)	1/296 (0.3)	
ICI treatment line			
First	260/505 (51.5)	166/296 (56.1)	.66
Second	163/505 (32.3)	89/296 (30.1)	
Third	57/505 (11.3)	31/296 (10.5)	
Fourth or later	25/505 (5.0)	10/296 (3.4)	
No. of cycles received, median (IQR)	4 (2-9)	10 (4-23)	NA
Best observed response^e^			
Complete response	3/315 (1.0)	1/255 (0.4)	<.001
Partial response	49/315 (15.6)	103/255 (40.4)	
Stable disease	128/315 (40.6)	111/255 (43.5)	
Progressive disease	135/315 (42.9)	40/255 (15.7)	

Abbreviations: BMI, body mass index (calculated as weight in kilograms divided by height in meters squared); DNLR, derived neutrophil-to-lymphocyte ratio; ECOG, Eastern Cooperative Oncology Group; ICI, immune checkpoint inhibitor; irAE, immune-related adverse event; NA, not applicable; PD-L1, programmed cell death ligand 1.

^a^ Unless otherwise indicated, data are expressed as No./total No. (%) of patients. Owing to missing data, total numbers may be less than numbers in column headings. Percentages have been rounded and may not total 100.

^b^ Two-tailed *P* ≤ .05 indicates statistical significance.

^c^ Lower scores indicate fewer restrictions and capable of more activity.

^d^ Patients may have multiple metastatic sites.

^e^ Best observed response was categorized per Response Evaluation Criteria in Solid Tumours, version 1.1.

Regarding the ICI agent, 509 patients received pembrolizumab (of whom 62 also received chemotherapy), 280 received nivolumab, and 9 received atezolizumab. Development of an irAE was not associated with the ICI agent or the treatment line. Whether the ICI was used as monotherapy or combined with chemotherapy was also not associated with developing an irAE. A complete or partial response to ICI treatment was associated with developing an irAE (104 of 255 [40.8%] vs 52 of 315 [16.5%]; *P* < .001).

### Characterization of irAEs

A total of 297 patients were diagnosed with a clinically meaningful irAE (37.0%). The median time to developing an irAE was 3.4 (IQR, 1.4-8.4) months. The most common irAEs were pneumonitis (82 [27.6%]), dermatitis (63 [21.2%]), and colitis (42 [14.1%]). Figure 1 further details the types of irAEs and their relative frequency per ICI agent used in our cohort.

No significant differences in the association of irAE relative frequencies were observed between individual ICI agents, or when ICI monotherapy was compared with ICIs combined with chemotherapy. The relative frequency of irAEs was 196 of 509 patients (38.5%) for pembrolizumab, 99 of 280 (35.4%) for nivolumab, 1 of 9 (11.1%) for atezolizumab, and 20 of 62 (32.3%) for ICI combined with chemotherapy.

During the follow-up period, nearly one-third of all clinically meaningful irAEs resulted in hospitalization (90 of 297 [30.3%]), with pneumonitis accounting for over half of hospitalized cases (47 of 90 [52.2%]). All patients with carditis were hospitalized (4 of 4 [100%]), followed in frequency by those with pneumonitis (47 of 82 [57.3%]) and adrenal insufficiency (4 of 8 [50.0%]). No significant differences in the association of hospitalization rates for irAEs were observed between individual ICI agents. The relative frequency of hospitalization for an irAE was 58 of 196 patients (29.6%) receiving pembrolizumab, 31 of 99 (31.3%) receiving nivolumab, and 1 of 1 (100%) receiving atezolizumab.

### Survival Outcomes and Time to Next Treatment After irAE Development and Hospitalization

**Figure 2.**
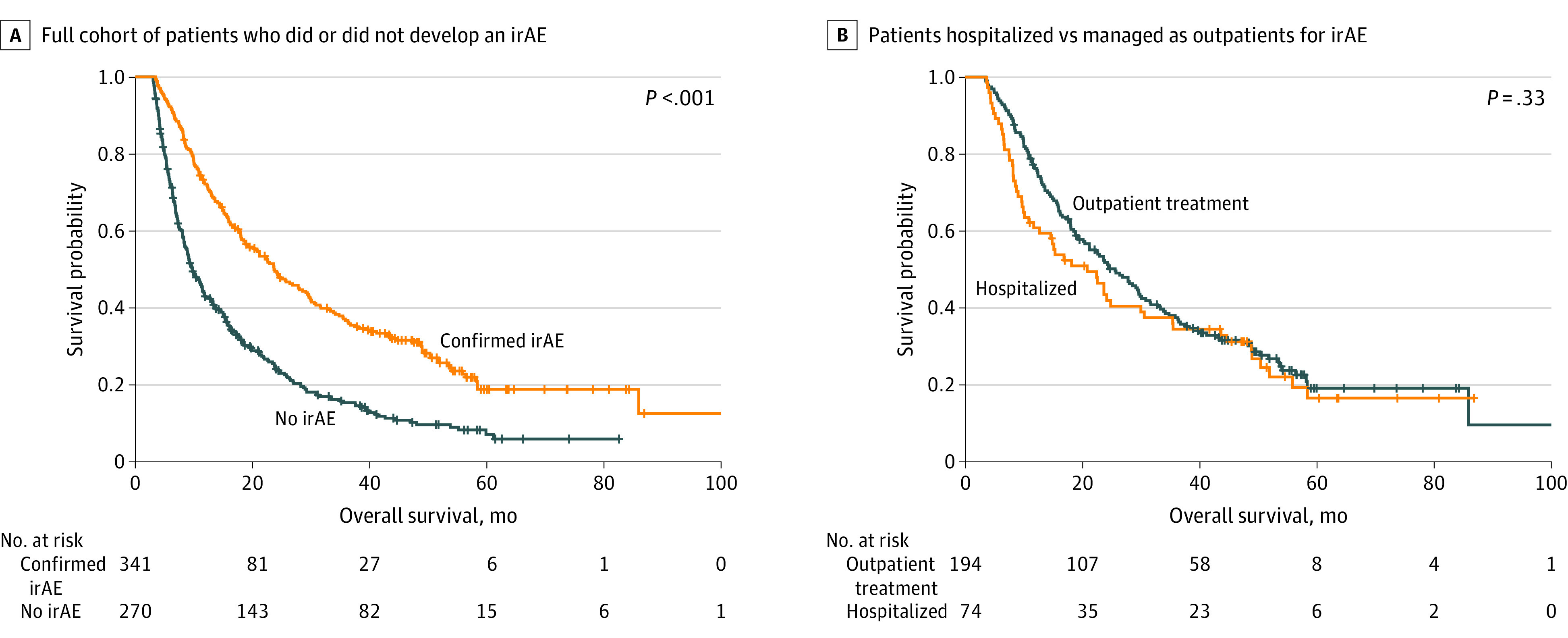
Median Overall Survival for Patients With Metastatic Non–Small Cell Lung Cancer Analysis includes patients who survived to at least 12 weeks. A, Full cohort of patients who did or did not develop an immune-related adverse event (irAE) (median OS, 23.7 [95% CI, 19.3-29.1] vs 9.8 months [95% CI, 8.7-11.4 months]; *P* < .001). B, Patients hospitalized compared with those treated as outpatients for irAEs (median OS, 20.8 [95% CI, 11.7-30.6] vs 25.6 [95% CI, 20.1-29.8] months; *P* = .33).

The median OS for the total cohort was 15.7 (95% CI, 13.0-16.6) months. In the 12-week landmark analysis (n = 611), developing a clinically meaningful irAE was associated with a significantly longer median OS compared with those patients who did not (23.7 [95% CI, 19.3-29.1] vs 9.8 [95% CI, 8.7-11.4] months; *P* < .001) (Figure 2A). For patients with a clinically meaningful irAE, no association with median OS was seen between those hospitalized and those treated as outpatients (20.8 [95% CI, 11.7-30.6] vs 25.6 [95% CI, 20.1-29.8] months; *P* = .33) (Figure 2B).

**Figure 3.**
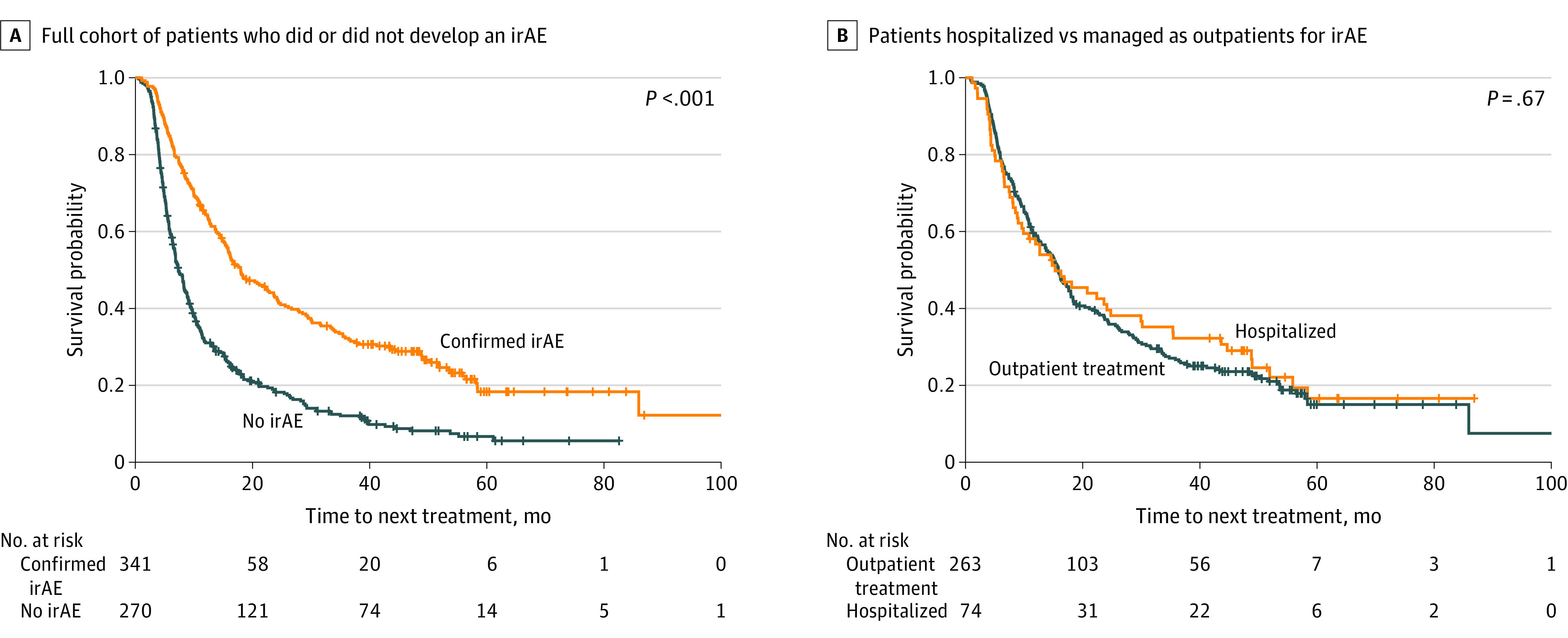
Median Time to Next Treatment for Patients With Metastatic Non–Small Cell Lung Cancer Analysis includes patients who survived to at least 12 weeks. A, Full cohort of patients who did or did not develop an immune-related adverse event (irAE) (median time to next treatment, 18.0 [95% CI, 15.6-22.9] vs 7.3 [95% CI, 6.6-8.4] months; *P* < .001). B, Patients hospitalized compared with those treated as outpatients for irAEs (median time to next treatment, 15.3 [95% CI, 9.6-24.8] vs 15.9 [95% CI, 13.6-18.0] months; *P* = .67).

Time to next treatment was the secondary outcome of interest. In the 12-week landmark analysis (n = 611), developing a clinically meaningful irAE was associated with a significantly longer median TTNT compared with those patients who did not (18.0 [95% CI, 15.6-22.9] vs 7.3 [95% CI, 6.6-8.4] months; *P* < .001) (Figure 3A). For patients with a clinically meaningful irAE, no association in median TTNT was seen between those hospitalized and those treated as outpatients (15.3 [95% CI, 9.6-24.8] vs 15.9 [95% CI, 13.6-18.0] months; *P* = .67) (Figure 3B).

### Variables Associated With OS by Cox Proportional Hazards Regression Analysis

To further evaluate the association between potential prognostic factors and OS among patients surviving to 12 weeks, Cox proportional hazards regression analysis was performed (Table 2). On multivariable analysis adjusted for all established prognostic factors (ECOG performance status, PD-L1 expression, organ sites of metastatic disease, DNLR, irAE development, and levels of hemoglobin, albumin, and LDH), developing an irAE remained independently associated with longer OS (hazard ratio, 0.53 [95% CI, 0.40-0.70]; *P* < .001).

**Table 2.** Multivariable Adjusted Cox Proportional Hazards Regression Analysis of Factors Associated With Overall Survival

Factor	Multivariable analysis	
	HR (95% CI)	*P* value^a^
irAE	0.53 (0.40-0.70)	<.001
Treatment line ICI		
Second (vs first)	1.27 (0.93-1.74)	.14
Third (vs first)	1.33 (0.80-2.21)	.27
Fourth (vs first)	1.77 (0.54-5.81)	.35
ECOG performance status at ICI therapy start		
1 (vs 0)	1.79 (1.24-2.60)	.002
2 (vs 0)	2.71 (1.68-4.35)	<.001
3 (vs 0)	4.18 (1.67-10.47)	.002
LDH level above reference range	1.23 (0.91-1.67)	.17
Albumin level below reference range	1.07 (0.78-1.46)	.68
Hemoglobin below reference range	0.82 (0.62-1.08)	.15
DNLR >3	1.19 (0.89-1.58)	.24
Histology		
Squamous cell carcinoma (vs adenocarcinoma)	1.31 (0.95-1.81)	.10
Other (vs adenocarcinoma)	1.44 (0.84-2.48)	.19
PD-L1 expression		
1%-49% (vs <1%)	1.06 (0.71-1.58)	.78
>50% (vs <1%)	0.64 (0.45-0.91)	.01
Metastatic site		
Bone	1.31 (0.97-1.77)	.08
Brain	1.28 (0.88-1.86)	.20
Liver	1.30 (0.91-1.86)	.15

Abbreviations: DNLR, derived neutrophil-to-lymphocyte ratio; ECOG, Eastern Cooperative Oncology Group; HR, hazard ratio; ICI, immune checkpoint inhibitor; irAE, immune-related adverse event; LDH, lactate dehydrogenase; PD-L1, programmed cell death ligand 1.

^a^ Two-tailed *P* ≤ .05 indicated statistical significance.

## Discussion

To our knowledge, this study represents the largest contemporary clinical dataset evaluating the association between irAEs and survival in locally advanced or metastatic NSCLC, agnostic to ICI agent, delivery as monotherapy or combined with chemotherapy, and treatment line. In our multicenter, retrospective population-based cohort, developing a clinically meaningful irAE was associated with longer OS in locally advanced or metastatic NSCLC. Association with improved OS continued among patients requiring hospitalization for irAE management.

It is clear ICI therapy is commonly complicated by irAEs. However, the mechanisms underlying irAE development are less clear, with 4 processes tentatively described: (1) increasing T-cell activity against antigens shared by tumor cells and healthy tissue; (2) accumulating levels of preexisting host autoantibodies; (3) rising levels of inflammatory cytokines; and (4) direct binding of anti–CTLA-4 antibodies with CTLA-4 found on healthy tissue, intensifying complement-mediated inflammation. Ultimately irAE development suggests the host immune system has been activated.^55^ Whether and how this translates to activation of antitumoral host immunity, with clinical outcomes subsequently affected, remains uncertain across solid malignant neoplasms. Cytokines, particularly interleukin 17, have been postulated to play a role in the antitumoral effects of ICIs, but further research is needed.^56,57,58^ Certainly, in our large cohort, irAE development was associated with improved OS. This supports previously published studies in NSCLC,^31,32,33,49,50,51^ recognizing they have largely been small, mainly examined nivolumab monotherapy, and inconsistently defined clinical impact of irAEs.

The role of irAE severity in the survival of patients with NSCLC remains contentious. This may reflect differences in toxicity grading, immunosuppressant use, and hospitalization thresholds. Like other published experiences,^32,33^ we found that irAE severity (reflected by management of toxic effects as an outpatient or hospitalized inpatient) did not compromise the survival gains seen with irAE development. However, this finding is not universally supported. A single study^52^ demonstrated only high-grade irAEs improve OS in NSCLC. In contrast, pooled data from phase 3 randomized clinical trials evaluating atezolizumab^31^ reported only low-grade irAEs are associated with improved survival in advanced NSCLC. Patients with high-grade irAEs had inferior survival except at a 12-month landmark analysis, where median OS was longer than among patients without irAEs.^31^ Improved OS being limited to patients with low-grade irAEs has been corroborated by several other groups^34,35,59^ and may be explained by the life-threatening potential of high-grade events. High-grade irAEs can result in death, albeit rarely. They may also require (permanent) treatment cessation and/or high-dose corticosteroids that can suppress antitumor efficacy of ICIs.^31,55^

The median time to developing an irAE in our study population was 3.4 months, longer than that typically cited in the NSCLC literature.^24,36,37,38^ However, we only recorded clinically meaningful irAEs per our definition, and studies have shown low-grade events develop earlier than high-grade events.^34,39^ The incidence of reported irAEs in NSCLC varies widely, ranging from 24.0% to 70.5%. This reflects heterogeneity including in study setting (observational vs randomized clinical trial), patient characteristics (cancer stage, PD-L1 status, geographical location, race and ethnicity), treatment parameters (ICI agent used either alone or combined with chemotherapy, treatment line), and irAE grading.^16,17,18,19,20,21,22,23,24,25,26,27,28,29,30,31,32,33,34,35,36,37,38,39,40,41,42,43,44,45,46,47,48^ In our cohort, the incidence of clinically meaningful irAEs was 37.0%. Nearly one-third of all clinically meaningful irAEs resulted in hospitalization (30.3%) for the management of toxic effects. Given our definition, grade 1 toxic effects, topically treated dermatitis, and mild hypothyroidism would not have been captured. Our incidence rates are thus best compared with irAEs higher than grade 2 and hospitalization rates with irAEs higher than grade 3.

Why irAEs occur in some patients but not others remains poorly understood, but a variety of predictive factors in NSCLC have been described. Demographic characteristics (age, sex, race, body mass index, and ECOG), concomitant medications (corticosteroids, proton pump inhibitors, and antibiotics), peripheral laboratory markers (hemoglobin, albumin, C-reactive protein, and others), tumor characteristics (histology, stage, PD-L1 expression, driver mutation status, and disease burden), and treatment-related factors (treatment line, response, ICI agent, concurrent chemotherapy, time to starting ICI, cumulative dose, and cumulative cycles of ICI) are inconsistently reported as predictive factors associated with irAE development.^16,17,18,19,20,21,22,23,24,25,26,27,28,29,30,48,60,61,62^ In our cohort, we identified several baseline patient characteristics associated with irAE development: 60 years or older, ECOG performance status 0, high expression of PD-L1, absence of bone metastases, DNLR of 3 or less, and levels of hemoglobin, albumin, and LDH within reference range. Other than response to ICI therapy, irAE development was not associated with treatment-related characteristics (ICI agent, ICI alone or in combination with chemotherapy, and treatment line) in our study population.

### Strengths and Limitations

Our study has several strengths. The sample size of 803 patients represents, to our knowledge, the largest contemporary clinical dataset examining irAEs and survival in locally advanced or metastatic NSCLC. As we used a 12-week landmark analysis, immortal time bias from patients with poor prognosis dying earlier than median onset of irAE development was minimized. Our multivariable Cox proportional hazards regression analysis adjusted for all established irAE predictive factors. Therefore, we can trust the association seen between irAE development and improved survival.

This study also has some limitations. How we defined an irAE differs from other studies, and focusing on clinically meaningful events may have influenced our results. We did not specifically capture grade 5 irAEs and thus could not differentiate death due to toxic effects compared with underlying disease in our 12-week landmark analysis. Although some patients may have died of grade 5 irAEs within 12 weeks, irAEs are very rarely fatal, so most excluded patients likely died of NSCLC.^63^ Where multiple irAEs occurred in the same patient, only the most clinically meaningful irAE was recorded. Thus, the association between the number of (clinically meaningful) irAEs and OS was not evaluated. We also did not evaluate the association between ICI rechallenge and OS. The existing evidence suggests rechallenge increases risk of further irAEs but, when compared with permanent ICI discontinuation, is not associated with survival.^64^ There are also limitations associated with retrospective studies, including unaccounted biases in patient selection, irAE capture and management, missing data, and incorporation of subjective assessments, including ECOG.

## Conclusions

In our large cohort of patients with locally advanced or metastatic NSCLC, developing a clinically meaningful irAE was associated with improved survival. This association was not compromised by hospitalization for management of severe toxic effects. Several patient baseline characteristics were associated with irAE development in our cohort, including age, ECOG performance status, sites of metastasis, PD-L1 expression, and laboratory parameters. Response to ICI therapy was the only treatment-related characteristic associated with irAE development.

## Notes

Supplement.
Data Sharing Statement
