# Supplementary material for: Immune-Related Adverse Events and Survival Among Patients With Metastatic NSCLC Treated With Immune Checkpoint Inhibitors
Source: JAMA Netw Open. 2024 Jan 18;7(1):e2352302. doi: 10.1001/jamanetworkopen.2023.52302 (PMC10797458; doi:10.1001/jamanetworkopen.2023.52302)
Supplement: Supplement. — Data Sharing Statement [file jamanetwopen-e2352302-s001.pdf]

Data Sharing Statement

Cook. Survival in Patients With Metastatic Non–Small Cell Lung Cancer Treated With Immune Checkpoint Inhibitors. *JAMA Netw Open*. Published January 18, 2024. doi:10.1001/jamanetworkopen.2023.52302

Data
Data available: No
